# Light-exposure at night impairs mouse ovary development via cell apoptosis and DNA damage

**DOI:** 10.1042/BSR20181464

**Published:** 2019-05-02

**Authors:** Yapeng Li, Shunfeng Cheng, Lan Li, Yong Zhao, Wei Shen, Xiaofeng Sun

**Affiliations:** 1 College of Animal Science and Technology, Qingdao Agricultural University, Qingdao 266109, China; 2 College of Life Sciences, Key Laboratory of Animal Reproduction and Germplasm Enhancement in Universities of Shandong, Qingdao Agricultural University, Qingdao 266109, China

**Keywords:** apoptosis, clock related genes, DNA damage, ovary, photoperiod

## Abstract

The alternation of light and dark rhythm causes a series of physiological, biochemical and metabolic changes in animals, which also alters the growth and development of animals, and feeding, migration, reproduction and other behavioral activities. In recent years, many studies have reported the effects of long-term (more than 6 weeks) illumination on ovarian growth and development. In the present study, we observed the damage, repair and apoptosis of ovarian DNA in a short period of illumination. The results showed that, in short time (less than 2 weeks) illumination conditions, the 24-h light treatment caused the reduction of total ovarian follicle number and down-regulation of circadian clock related genes. Furthermore, the changed levels of serum sex hormones were also detected after 24-h light exposure, of which the concentrations of LH (luteinizing hormone), FSH (follicle-stimulating hormone) and E2 (estradiol) were increased, but the concentration of PROG (progesterone) was decreased. Moreover, 24-h light exposure increased the expression of DNA damage and repair related genes, the number of TUNEL and RAD51 positive cells. These results indicated that 24-h light exposure for 4, 8 and 12 days increased DNA damage and cell apoptosis, thereby affecting the development of ovary.

## Introduction

It is well known that photoperiod affects the physiological behavior of animal by adjusting the daily and seasonal changes, and the animals develop specific adaptive periodic activities through rhythm oscillation [1–4]. Many studies have found that the photoperiod regulates animal body, and animals have to adapt to the daily and seasonal changes through the circadian oscillator of hypothalamic suprachiasmatic nucleus. It has been proved that biological rhythms can be determined or regulated by clock genes. And the expression of clock genes in the putative hypothalamus and its suprachiasmatic nucleus varies with the photoperiod or the light condition [5,6]. In mammals, several biological clock genes important for the circadian rhythms have been identified, such as period genes (*Per1, Per2*), cryptochrome genes (*Cry1, Cry2*) and RAR-related orphan receptors genes (*Rorα, Rorβ, Rorγ*) [7–9]. *Per* is the first clock gene discovered by Konopka and Benzer in 1971. Different point mutations within *Per* coding region can lengthen, shorten or abolish circadian rhythms [10]. Moreover, *Per* gene overexpression could restore the behavior of circadian rhythms caused by *Per* gene mutated [11], this further showed the important roles of *Per* gene in generation and maintenance of biological rhythms [12]. In the core feedback loop, transcriptional activator BMAL1 and CLOCK heterodimer initiates the transcription of *Per* and *Cry* genes, which are then suppressed by their products of translation and translocation [7,13,14].

Through the rhythm oscillator, photoperiod can adjust downstream genes’ expression of organisms [6,15]. And the daily physiological rhythm disorder can cause many pathophysiological issues, such as cognitive disturbance, mental illness and so on [16–21]. It was hypothesized that the biological clock participated in the daily length measurement [22]. Several groups have investigated the expression of photoperiod-induced ovarian circadian clock genes to figure out the effects of photoperiod on biological clock and ovarian development [7,23,24].

In mammals, the development of ovarian follicles consists of serial highly orchestrated events [25–27]. The interaction between oocytes and surrounding somatic cells is particularly important for the development and function of ovaries [28–31]. These interactions regulate the occurrence of germ cells, meiosis and the formation of primordial follicles, and then regulate follicle growth, maturity, ovulation and other processes [32–34]. As a daily and seasonal oscillatory rhythm, photoperiod can alter ovarian development cycle, inhibit or delay the sex maturation of different species [35–37]. At the same time, the endocrine environment and related factors can also be changed by photoperiod, such as changes in the relevant hormone content [38,39]. Light as one of the key external environmental cues can affect the circadian rhythms [40]. Many previous studies have reported that compared with normal light cycle, both increasing the light and decreasing the light exposure can affect ovarian follicular development [41–44]. These investigations suggest that photoperiod and other related signaling pathways affect the development of reproductive system, the secretion of sex hormones.

Even though many studies have presented the effects on ovarian growth and development caused by long-term (more than 6 weeks) illumination [45,46], the effects of the short period of illumination on ovary development have not been thoroughly investigated, and the underlying mechanism remains unclear. Therefore, the purpose of this investigation was to explore the effects of short period of illumination on DNA damage/repairment, and apoptosis in the mouse ovary.

## Materials and methods

### Animals and experiment design

All procedures of animal handing were approved by the Ethics Committee of Qingdao Agriculture University, Shandong, China [47]. Puberty CD-1 mice (4 weeks old) used in this experiment were purchased from Vital River Laboratory Animal Technology Co. LTD (Beijing, China). They were housed in temperature controlled (21–22°C) and standard diet conditions. 120 mice were randomly divided into two groups. One group was maintained under 24:0 h light/dark cycle (24-h light exposure), and the other was maintained under 16:8 h light/dark cycle (16-h light exposure) [48] as control. The light intensity is about 250 lx. When they were treated for 4, 8 and 12 days, respectively, the mice were euthanized and sampled for further analysis at 4 pm.

### RNA extraction, cDNA synthesis and quantitative RT-PCR

Ovaries of mice under different light-dark cycles (exposed for 4, 8 and 12 days) were collected. Total RNA was extracted using RNAprep pure MicroKit (Aidlab, RN07, China). Reverse transcription was performed using TURE script first strand cDNA Synthesis Kit (Aidlab, PC1802). Quantitative real-time PCR (qRT-PCR) was carried out using Light-Cycler^®^ SYBR Green I Master Kit (Roche, 04887352001, Switzerland) with a Roche real time PCR instrument (Roche LC480) according to the manufacturer’s instructions. qRT-PCR primers were listed in Table 1. The PCR reaction programs were set as follows: 10 min at 95°C, followed by 45 cycles of 95°C for 10 s, 60°C for 30 s and 72°C for 20 s. The relative mRNA abundance was normalized to the expression of *β-actin* according to following formula: 2^−(target gene *C*^_T_^ value − reference gene *C*^_T_^ value)^. Data represented were calculated by mean ± standard deviation (S.D.) of at least three independent experiments.

**Table 1:** Primers used for qRT-PCR

Genes	Sequences of primers	Production (bp)
*β-Actin*	F: TCGTGGGCCGCTCTAGGCAC; R: TGGCCTTAGGGTTCAGGGGG	255
*Rorα*	F: CCCCTACTGTTCCTTCACCA; R: CCAGGTGGGATTTGGATATG	95
*Rorβ*	F: TAGCTCCCGGGATAACAATG; R: GCCAGCTGATGGAGTTCTTC	106
*Rorγ*	F: TGCAAGACTCATCGACAAGG; R: AGGGGATTCAACATCAGTGC	177
*Dbp*	F: CGTGGAGGTGCTTAATGACCTTT; R: ATGGCCTGGAATGCTTGA	68
*Bmal1*	F: CCAAGAAAGTATGGACACAGACAAA; R: GCATTCTTGATCCTTCCTTGGT	81
*Cry1*	F: CTGGCGTGGAAGTCATCGT; R: CTGTCCGCCATTGAGTTCTATG	77
*Per1*	F: ACCAGCGTGTCATGATGACATAC; R: CTCTCCCGGTCTTGCTTCAG	73
*Per2*	F: ATGCTCGCCATCCACAAGA; R: GCGGAATCGAATGGGAGAAT	72
*Ck1ε*	F: CGGTTCGATGATAAGCCTGACT; R: AAACCCTGCCGGTGAAAGA	71
*Rad51*	F: ACCAGACCCAGCTCCTTTAC; R: CAAGTCGAAGCAGCATCCTC	171
*BRCA1*	F: ATCCCGGGAAAAGCTCTTCA; R: GGCTGCACGATCACAACTAG	171
*Rec8*	F: TGATATGGAGGAGGCTGACC; R: GCAGCCTCTAAAAGGTGTCG	165

### Immunofluorescence and immunohistochemistry

For immunofluorescence, ovaries from 24-h light exposed mice and the control group were collected and fixed in 4% paraformaldehyde (Solarbio, P1110, Beijing, China) for 12 h. Next, they were washed with running water, and dehydrated by gradient alcohol and embedded in paraffin. After the ovaries were cut into 5-μm serial sections, they were heated at 60°C for 2 h, dewaxed by xylene and rehydrated by gradient alcohol, Antigen retrieval was performed with trisodium citrate at the condition of 96°C for 10 min. After cooled to room temperature, the ovary slides were blocked with BDT (3% BSA and 10% goat serum dissolved in TBS) for 30 min, then incubated with anti-RAD51 (Abcam, ab88572, U.S.A.) primary antibody at a dilution of 1:1000 overnight at 4°C. The next day, the sections were incubated with Cy3-conjugated goat anti-mouse secondary antibody (Beyotime, A0521, Nantong, China) at a dilution of 1:2000 for 30 min. Finally, Hoechst 33342 was used for nuclear staining. Follicles were amounted and scored as previously described [49]. One out of every four sections was selected from the whole-ovary serial sections for analyzing the percentage of RAD51 positive cells (the number of RAD 51 positive cells/the total number of cells).

For immunohistochemical staining, 3% H_2_O_2_ was used to eliminate endogenous peroxidase. Then the sections were incubated with anti-VASA polyclonal antibody at a dilution of 1:1000 overnight at 4°C (Abcam, ab13840). After washing, the sections were incubated with HRP-labeled goat anti-rabbit secondary antibody at a dilution of 1:2000 for 30 min (Beyotime, A0208). After that, the color was developed using DAB peroxidase (Beyotime, P0203) and the sections were counterstained with hematoxylin. All ovaries slices were photographed under Olympus microscope (BX51, Japan). The positive rates were analyzed using Image Plus software, and finally visualized by GraphPad Prism 5. Each immunofluorescence or immunohistochemistry were repeated at least three times.

### TUNEL staining

Parallel to procedure stated in immunofluorescence or immunohistochemistry staining, ovary slides were dewaxed, rehydrated. Then TUNEL BrightRed Apoptosis Detection Kit (Vazyme, A113, Nanjing, China) was used to evaluate ovarian cell apoptosis according to the manufacturer’s instructions. After treated with proteinase for 20 min at room temperature, ovary sections were incubated with TUNEL reaction mixture (Label Solution and Enzyme Solution; 5:1) for 60 min at 37°C and stained with Hoechst33342. The sections were observed and photographed under BX51 Olympus fluorescence microscope. One out of every four sections was selected from the whole-ovary serial sections for analyzing the percentage of TUNEL positive cells (the number of TUNEL positive cells/the total number of cells).

### Western blotting

Western blotting analysis of ovary protein lysates was based on procedure represented previously [50,51]. Proteins from ovaries of different treatment were separated and transferred onto PVDF membranes. After blocking with 5% BSA in Tris-buffer, the membranes were incubated with anti-RAD51 antibody (Abcam, ab88572) and anti-ACTIN antibody (Abcam, ab8226) at a concentration of 1.0 μg/ml overnight at 4°C. Then the membranes were incubated at 37°C for 2 h with secondary antibodies (Beyotime, A0216) at a dilution of 1:2000. Proteins were detected using the BeyoECL Plus Kit (Beyotime, P0018). The band intensity was qualified with alphaview software.

### Levels of luteinizing hormone, follicle-stimulating hormone, estradiol and progesterone

After treatment, the blood samples were collected from the retroorbital sinus immediately. Then the blood samples were centrifuged at 3000 rpm. for 30 min to obtain the serum. The serum samples were quickly removed and kept at −80°C for subsequent analysis of hormone levels. The serum sex hormone levels were measured using (progesterone) PROG ELISA kit (JingmaBIO, E-20375, China), (follicle-stimulating hormone) FSH ELISA kit (JingmaBIO, E-20418), (luteinizing hormone) LH ELISA kit (JingmaBIO, E-20342) and (estradiol) E2 ELISA kit (JingmaBIO, E-20380) according to the manufacturer’s instructions. Each experiment was carried out in triplicate.

### Statistics

All data represent the mean ± S.D. at least three independent experiments. Statistics difference was determined by Student’s *t*-test or one-way ANOVA with GraphPad Prism 5. Results were considered statistically significant at *P*<0.05.

## Results

### 24-h light exposure reduced the number of follicles

**Figure 1:**
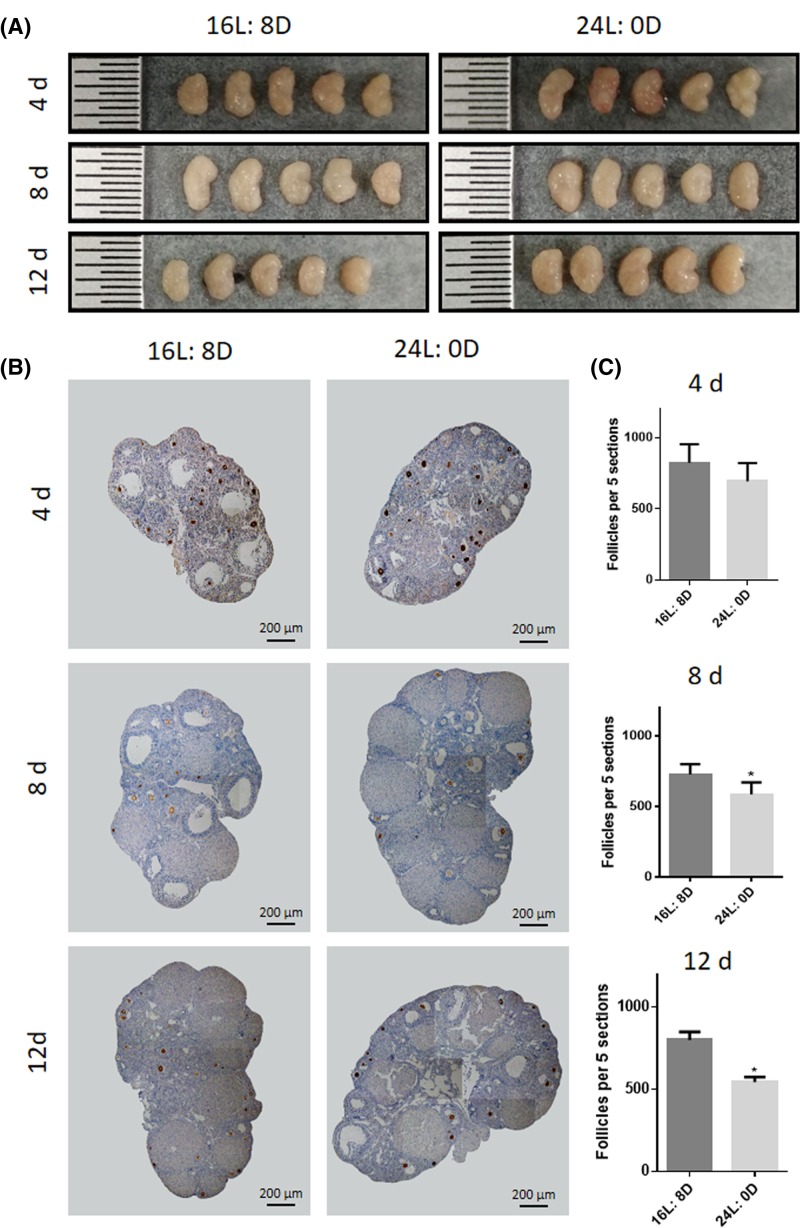
Effects of photoperiod exposure on ovarian development (**A**) Ovarian size and morphology, (**B**) Immunohistochemical staining for VASA, (**C**) the number of follicles. Data are expressed as mean ± S.D. at least three independent experiments (^*^P<0.05).

Though there were no significant changes in ovarian morphology and size after 24-h light exposure for 4, 8 and 12 days (Figure 1A), the number of oocytes in 24-h light exposure group significantly decreased after 8-day treatment compared with that in control group by staining with oocyte specific marker VASA (^*^P<0.05; Figure 1B,C).

### Photoperiod altered serum sex hormone levels

**Figure 2:**
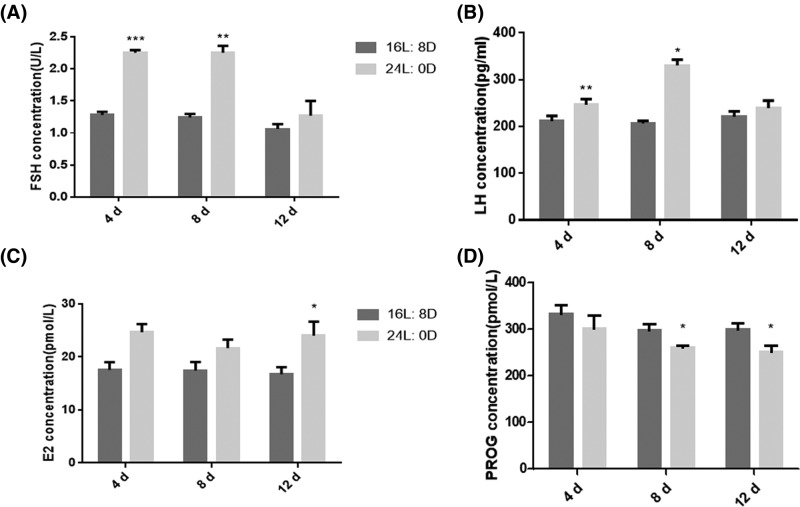
Effects of photoperiod exposure on serum sex hormone levels (**A**) FSH, (**B**) LH, (**C**) E2, (**D**) PROG. Data are expressed as mean ± S.D. at least three independent experiments (^*^P<0.05, ^**^P<0.01).

Based on the methods mentioned above, we speculated that photoperiod might regulate the ovarian development and follicle number by influencing serum sex hormone levels. Therefore, we examined the levels of sex hormones in serum. The results showed that, compared with that in control group, 24-h light exposure significantly increased the levels of FSH (Figure 2A), LH (Figure 2B) after 4- and 8-day treatment, and the level of E2 (Figure 2C) after 12-day treatment, however, significantly decreased PROG concentration (Figure 2D) after 8- and 12-day treatment. Meanwhile, we chose several mice of each group to examine the estrous cycles based on a smear test; however, there was no significant difference between them (data not shown).

### 24-h light exposure increased the apoptosis of ovarian cells

**Figure 3:**
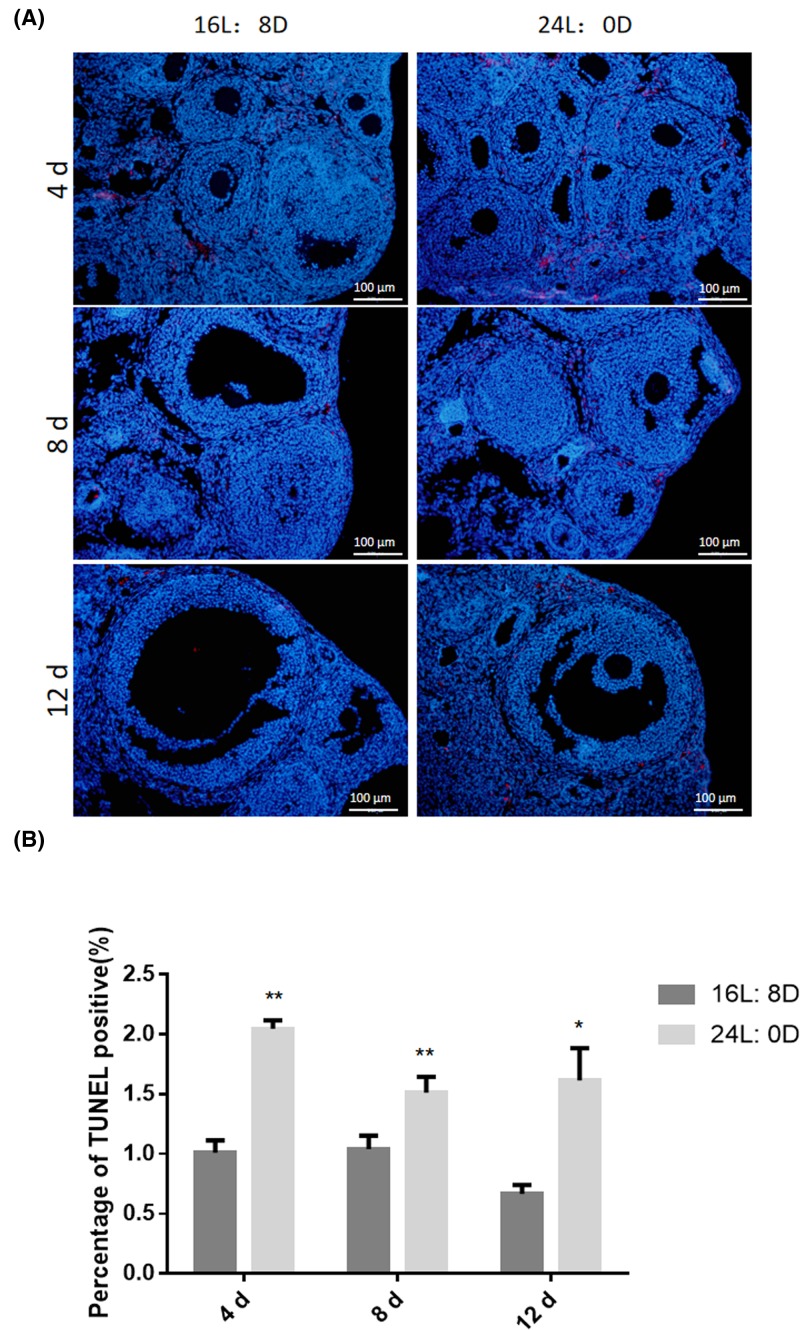
Effects of photoperiod exposure on cell apoptosis (**A**) TUNEL immunostaining of ovaries. (**B**) Quantification of the percentage of TUNEL positive cells. Data are expressed as mean ± S.D. at least three independent experiments (^*^P<0.05, ^**^P<0.01).

The positive number of ovarian apoptotic cells in the 24-h light exposure group and control group were analyzed by TUNEL staining (Figure 3A). Compared with the control group, 24-h light exposure for 4, 8 and 12 days significantly increased the number of TUNEL positive somatic cells in ovaries (^*^P<0.05 or ^**^P<0.01; Figure 3B).

### 24-h light exposure decreased the expression of murine ovary circadian clock-related genes

**Figure 4:**
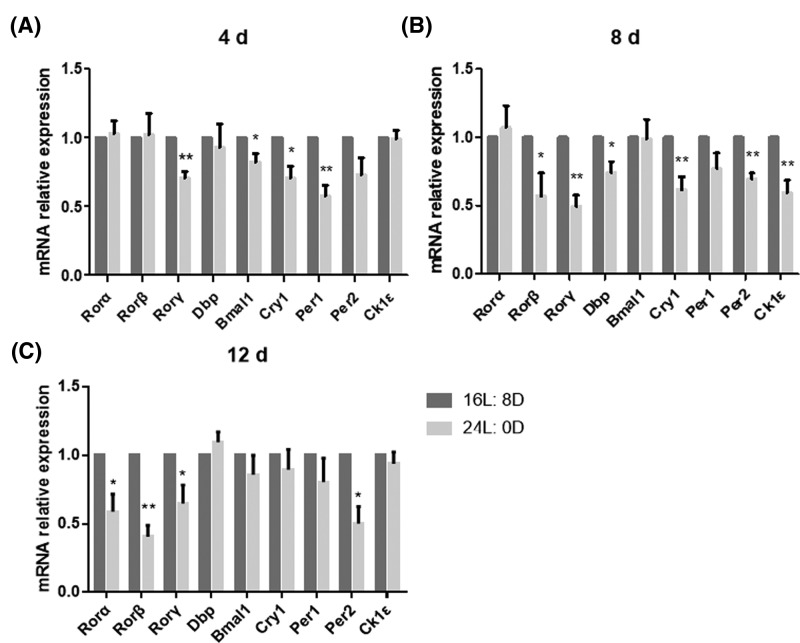
Effects of photoperiod exposure on mRNA levels of ovarian circadian clock related genes (**A**) Treated for 4 days, (**B**) Treated for 8 days and (**C**) Treated for 12 days. Data are expressed as mean ± S.D. at least three independent experiments (^*^P<0.05, ^**^P<0.01).

After 4 days’ treatment, we found that 24-h light exposure significantly reduced the expression of *Rorγ, Bmal1, Cry1* and *Per1* (^*^P<0.05 or ^**^P<0.01) in mRNA level compared with that in control group (Figure 4A). The expression of *Rorα, Rorβ, Rorγ, Per2* and *Ck1ε* was significantly decreased after 8 days’ treatment (^*^P<0.05 or ^**^P<0.01; Figure 4B), and the expression of *Rorα, Rorβ, Rorγ, Per2* and other genes was significantly decreased after 12 days’ treatment compared with that in control group (^*^P<0.05 or ^**^P<0.01; Figure 4C).

### 24-h light exposure increased ovarian DNA damage

**Figure 5:**
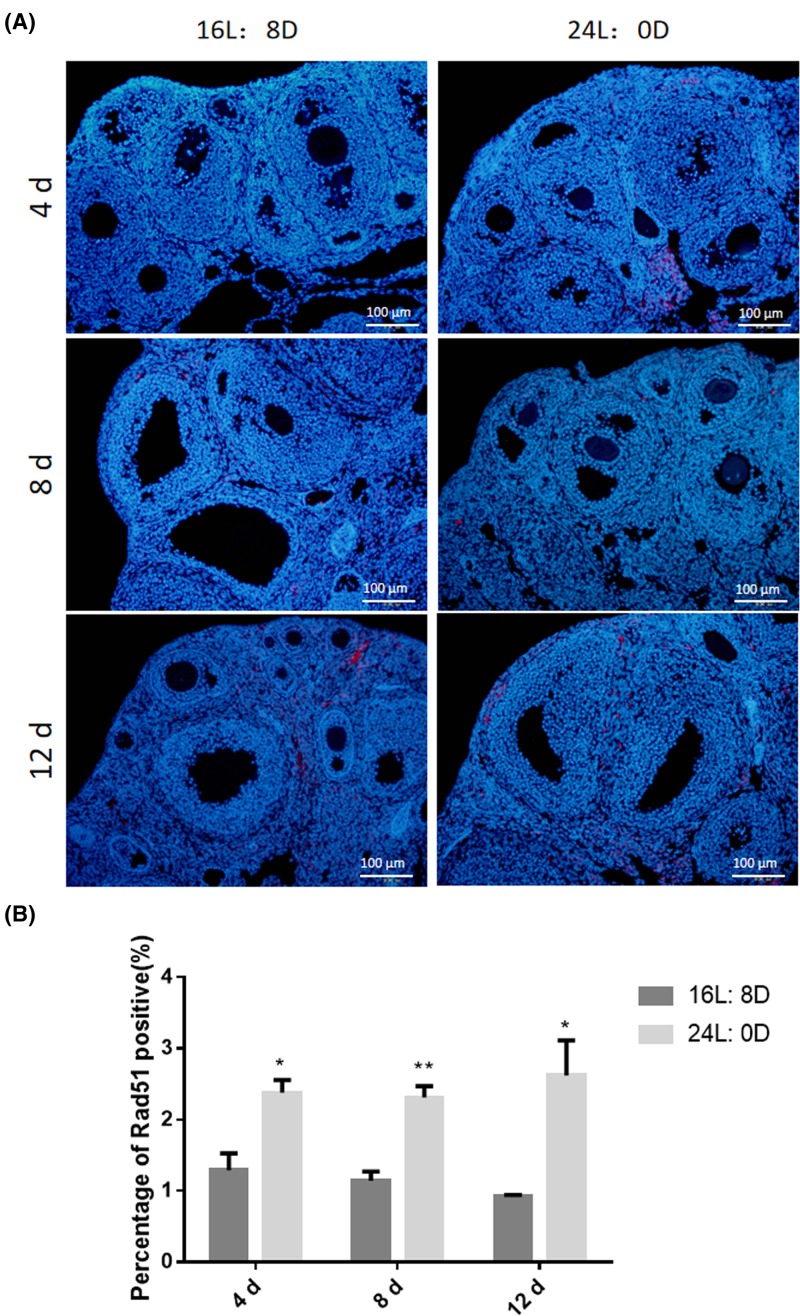
Effects of photoperiod exposure on Rad51 expression (**A**) Immunoflurescence of RAD51, (**B**) Quantification of percentage of RAD51 positive cells. Data are expressed as mean ± S.D. at least three independent experiments (^*^P<0.05, ^**^P<0.01).

**Figure 6:**
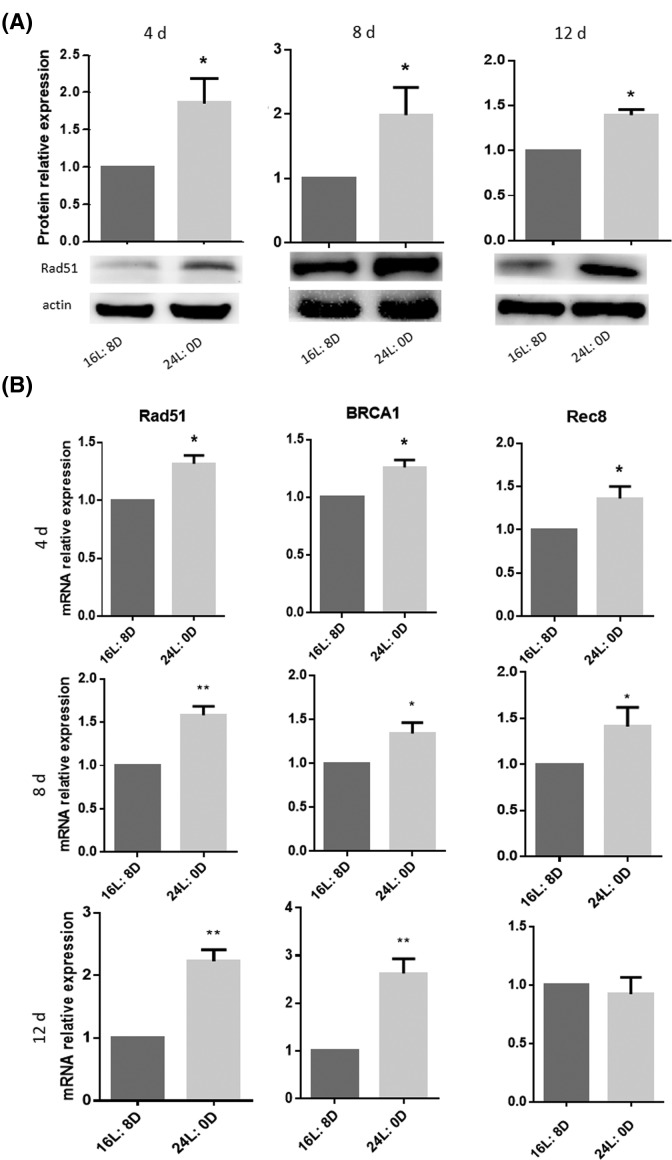
Effects of photoperiod exposure on DNA damage and repair related genes (**A**) Protein level of Rad51 by Western blot, (**B**) The mRNA levels of Rad51, BRCA1 and Rec8 by RT-qPCR. Data are expressed as mean ± S.D. at least three independent experiments (^*^P<0.05, ^**^P<0.01).

To investigate the mechanism of ovarian cell apoptosis, the DNA damage-related protein RAD51 was detected using immunofluorescence and Western blot. The number of RAD51 positive somatic cells in the ovaries was increased significantly after 24-h light exposure for 4, 8 and 12 days compared with that in control group (^*^P<0.05 or ^**^P<0.01; Figure 5A,B). Furthermore, Western blot results also proved the increased expression of RAD51 (^*^P<0.05; Figure 6A). Then, the expression of DNA damage and repairment related genes *Rad51, BRCA1* and *Rec8* were detected significantly up-regulated at mRNA level after 24-h light exposure for 4, 8 and 12 days compared with that in control group (^*^P<0.05 or ^**^P<0.01; Figure 6B).

## Discussion

There have been many studies reported that long-term (more than 6 weeks) exposure to light affected ovarian development in mice, rats, cattle, sheep and other mammals [52–55]. The reproductive cycle is related to the seasonal variation of the photoperiod [56–58], and the prolongation of the artificial photoperiod can affect ovarian development, which is based on the reflect of circadian rhythm to light [8,59,60].

In the present study, we focused on the effects of short term (less than 2 weeks) exposure to 24-h light on ovarian development. In order to avoid the effects of temporally cycling on the level of sexual hormones and clock gene expression, the sampling timing was consistently at 4 pm. Though, there was no significant change in the food intake, and the body weight gain after short term (less than 2 weeks) exposure to light at nights (data not shown), the number of ovarian follicles was decreased. Especially, there was a significant decrease after 8- and 12-day exposure. The relationship between this significant change in the number of follicles and the change in the body weight increasing rate remains to be further studied. We speculate that some of these results may be related to DNA damage repair caused by short-term treatment of 24-h light exposure.

We assume that this periodicity of the photoperiod is closely related to the reproductive cycle of the female. In terms of the levels of serum hormones, our results showed that short-term (less than 2 weeks) exposure to light at night increased the levels of LH, FSH and E2, but decreased the level of PROG. In the present study, large numbers (120 mice) of samples were used and were grouped randomly to exclude the effects of estrus cycle on sex hormones. And the changes of the sex hormones tested were significant, which suggested that the light cycle had a strong interference with reproductive hormones or the reproductive cycle. On the other hand, the changes of hormone level might be one of the reasons for the decreased number of follicles caused by prolonged photoperiodism. In addition, we found that short-term (less than 2 weeks) exposure to light at night increased ovarian apoptosis and DNA damage. It suggested that the short-term (less than 2 weeks) exposure to light at nights might alter the level of sex hormones and induce cell apoptosis and DNA damage through neurohormonal regulation, thus affected follicular development; however, the specific regulatory mechanisms remain to be further studied.

It is generally considered that photoperiod exerted its function of neurohumoral regulation via the suprachiasmatic nucleus of mammalian hypothalamus. Based on this, we verified the expression of some genes related to the circadian clock. For a long time, *Per* was the only circadian gene known, but rapid progress in the circadian field led to the identification of other clock genes [40]. Now, it is known that many clock genes were found to play roles in controlling or regulating biological rhythm, which were also found expressed in animal ovaries [61]. In the present study, we also detected the mRNA expression level of clock genes and found that some of detected biological clock genes significantly decreased after 24-h light exposure for 4, 8 or 12 days. The results showed that the altered circadian rhythm had affected the expression of clock genes, and suggested a potential neuromodulatory effect of the genes associated with peripheral ovarian clocks.

It is well known that environmental toxins, UV and ionizing irradiation usually can induce DNA damage [62]. Light-exposure at night, regard as one of the genotoxic stresses, can disrupt DNA damage response and repair [63]. DNA damage-induced cell apoptosis has been well studied from the year of 2000 [64], in which DNA damage can active DNA repair pathways. If the damage is above the competence of the repair, the cells enter the program of apoptosis and undergo apoptosis. Many reports also confirmed that sex hormones played roles in cell apoptosis [65,66]. Some hormones act as survival factors to inhibit apoptosis and others act as atretogenic factors to induce apoptosis [67–70]. According to our results, 24-h ight exposure for less than 2 weeks could induce the reproductive hormones disorder, thereby affecting cell apoptosis. Or 24-h ight exposure as one of the genotoxic stresses induced DNA damage then resulted in DNA damage-induced cell apoptosis. The increasing apoptosis was responsible for the number of follicles and the development of ovary.

However, the oscillation phase should be considered in studying the issues of circadian changes. Whether the oscillation phase in sexual hormone secretion or clock gene expression is synchronized or not and whether the oscillation phase in sexual hormone secretion or clock gene expression can be changed by the treatment of the present study will be further explored.

## Abbreviations

DAB: 3,3-diaminobenzidine
E2: estradiol
FSH: follicle-stimulating hormone
HRP: horseradish peroxidase
LH: luteinizing hormone
PROG: progesterone
qRT-PCR: quantitative real-time PCR
TBS: tris buffered saline
